# Metal–organic framework–derived Ni@C and NiO@C as anode catalysts for urea fuel cells

**DOI:** 10.1038/s41598-019-57139-7

**Published:** 2020-01-14

**Authors:** Thao Quynh Ngan Tran, Bang Ju Park, Woo Hyun Yun, Tien Nhac Duong, Hyon Hee Yoon

**Affiliations:** 1 Department of Machine and Equipment, Industrial University of Ho Chi Minh City, Nguyen Van Bao, HCMC Vietnam; 2 Department of Electronic Engineering, Gachon University, 1342 Seongnam-daero, Seongnam, Gyeonggi-do 13120 Republic of Korea; 3 Department of Chemical and Bio Engineering, Gachon University, 1342 Seongnam-daero, Seongnam, Gyeonggi-do 13120 Republic of Korea

**Keywords:** Electrocatalysis, Fuel cells, Metal-organic frameworks

## Abstract

Highly porous self-assembled nanostructured Ni@C and NiO@C were synthesized via calcination of a Ni-based metal–organic framework. The morphology, structure, and composition of as synthesized Ni@C and NiO@C were characterized by SEM, FIB-SEM, TEM, and XRD. The electro-catalytic activity of the Ni@C and NiO@C catalysts towards urea oxidation was investigated using cyclic voltammetry. It was found that the Ni@C had a higher residual carbon content and a higher specific surface area than NiO@C, thus exhibiting an enhanced electrochemical performance for urea oxidation. A direct urea fuel cell with Ni@C as an anode catalyst featured an excellent maximum power density of 13.8 mW cm^−2^ with 0.33 M urea solution in 1 M KOH as fuel and humidified air as oxidant at 50 °C, additionally showing excellent stability during continuous 20-h operation. Thus, this work showed that the highly porous carbon-supported Ni catalysts derived from Ni-based metal–organic framework can be used for urea oxidation and as an efficient anode material for urea fuel cells.

## Introduction

The non-toxicity, affordability, and ease of storage/transport of urea make direct urea fuel cells (DUFCs) promising power sources^1,2^. The synthesis of urea features the reaction of ammonia with carbon dioxide, thus simultaneously achieving carbon dioxide fixation and the conversion of toxic ammonia into a non-toxic product^2^. Moreover, DUFCs can be operated using urine or urea-rich wastewater as fuels. Although the high energy density (16.9 MJ L^−1^) and high solubility of urea allow its use as a hydrogen carrier in solid oxide fuel cells^3^, its direct utilization for electrical energy production is believed to bring about further system simplification and increase the energy conversion efficiency^4^. In a urea/O_2_ type DUFC, urea is electro-oxidized at the anode (Eq. 1), while oxygen is reduced at the cathode (Eq. 2):

1 $${\rm{Anode}}:{\rm{CO}}{({{\rm{NH}}}_{2})}_{2}+6{{\rm{OH}}}^{-}\to {{\rm{N}}}_{2}+{{\rm{CO}}}_{2}+5{{\rm{H}}}_{2}{\rm{O}}+6{e}^{-},E^\circ =-0.746\,{\rm{V}}$$

2 $${\rm{Cathode}}:{{\rm{O}}}_{2}+2{{\rm{H}}}_{2}{\rm{O}}+4{e}^{\mbox{--}}\to 4{{\rm{OH}}}^{\mbox{--}},E^\circ =+0.401$$

3 $${\rm{Overall}}:2{\rm{CO}}{({{\rm{NH}}}_{2})}_{2}+3{{\rm{O}}}_{2}\to 2{{\rm{N}}}_{2}+2{{\rm{CO}}}_{2}+4{{\rm{H}}}_{2}{\rm{O}},E^\circ =+\,1.147\,{\rm{V}}$$

Although the theoretical open circuit voltage (OCV) of a urea/O_2_ fuel cell equals 1.147 V at room temperature which is lower than that of a H_2_/O_2_ fuel cell (1.23 V)^5^, the theoretical efficiency of the former cell is ~20% higher than that of the latter^6^ due to the positive entropy change of the overall reaction (Eq. 3). In addition, DUFCs can be operated using anion exchange membranes and catalysts based on non-precious metal such as Ni that exhibit good activity in alkaline environments^3^. However, the practical implementation of DUFCs is hindered by their relatively low power density of 5–10 mW cm^−2^ compared to H_2_ and CH_3_OH fueled anion exchange membrane fuel cells which have typical maximum power densities of 500–600 and 100–300 mW cm^2^, respectively^3,7,8^. Hence, most research efforts are directed at the development of suitable anode catalysts to speed up the sluggish kinetics of the six-electron-transfer urea oxidation reaction (UOR). Currently, non-precious Ni is considered to be the most efficient anode catalyst^9,10^ in DUFCs. The activity and stability of Ni-based catalysts have been improved by doping with various transient metals such as Co, Mo, Cr, Mn, and Fe^11–14^. In addition, nanostructured Ni catalysts (e.g., those featuring nanowires^10,15^, nanosheets^9,16^, and nanoparticles^17,18^) and/or composition with carbon-based nanostructures including carbon nanotubes^19,20^, graphene^17^, and aerogels^21^ are reported to exhibit increased numbers of active sites, electrical connections, porosity, and stability.

Recently, the highly tunable porosity, large surface area, and open metal sites of metal-organic frameworks (MOFs) have attracted much attention, allowing MOFs to be viewed as excellent electrochemically functional materials^22^. For instance, we have previously demonstrated the excellent catalytic activity of a Ni-based MOF (Ni-MOF) for urea oxidation^20^. However, the electrical conductivity of Ni-MOFs is generally poor, requiring hybridization with highly conductive compounds such as carbon nanotubes to obtain high urea oxidation currents^20^. Furthermore, the implementation of MOFs as electrochemical catalysts is restricted by their weak mechanical stability^23–25^. Ni-MOF has been pyrolyzed to obtain NiO@C for lithium storage. The NiO@C was reported to exhibit a hierarchical core-shell structure and feature a large surface area and high porosity^26^ due to the intrinsic pore structure of the MOF precursor^27^. Additionally, an iron-based MOF has been utilized to prepare a spindle-like porous α-Fe_2_O_3_ electrocatalyst^28^.

In this study, we pyrolized Ni-MOF under air and N_2_ environment to obtain novel NiO@C and Ni@C catalysts, respectively, for UOR. The structural, morphological, and electrochemical properties of the prepared NiO@C and Ni@C catalysts were characterized. The performance of DUFCs (urea/O_2_ fuel cells) comprising the NiO@C and Ni@C catalysts as anode materials was evaluated.

## Results and Discussion

### Characterization of Ni-MOF, Ni@C, and NiO@C

**Figure 1:**
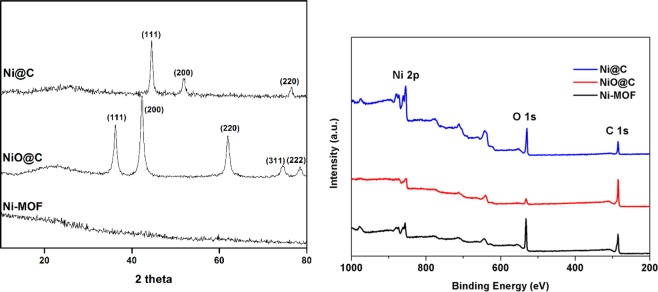
(**a**) XRD patterns of Ni-MOF, Ni@C, and NiO@C and (**b**) XPS spectra of Ni-MOF, NiO@C, and Ni@C.

The crystallographic structure and phase purity of the Ni-MOF precursor, Ni@C, and NiO@C powders were analyzed by XRD (Fig. 1a). Ni-MOF exhibited no appreciable crystalline phase peaks, which indicated its amorphous nature and was in agreement with previous reports^20,26^. In contrast, the patterns of Ni@C and NiO@C showed peaks of metallic Ni [(111), (200), (220); JCPDF No. 65-0380]^29^ and NiO [(111), (220), (220), (311), (222), (225); JCPDF No. 65-2901]^26^, confirming the successful synthesis of the above catalysts. Moreover, the absence of any impurity peaks in the above patterns confirmed that the thermal decomposition of Ni-MOF was complete and afforded phase-pure Ni and NiO. In addition, the co-presence of carbon in Ni@C and NiO@C was confirmed by the observation of a peak at 2*θ* = 23.2°^30^. The presence of residual carbon was thought to account for the self-assembled hierarchical structure of Ni@C and NiO@C, as is further discussed later in the text. The carbon content of Ni@C (calcined in N_2_) and NiO@C (calcined in air) were 27.0 and 0.2 wt%, respectively, as measured by an elemental analyzer (Table 1). The difference in carbon content of Ni@C and NiO@C was close to the difference in remaining weight of each sample as shown in TG analysis (Suppl. Fig. S1) due to the removal of carbon by CO_2_ formation during the calcination in air environment. The XPS spectra of Ni-MOF, NiO@C and Ni@C are presented in Fig. 1b, indicating the presence of Ni, carbon and oxygen. The deconvulated core level spectra of the different materials shows two distinct peaks corresponding to Ni 2p_3/2_ and Ni 2p_1/2_ (Suppl. Fig. S2). In case of Ni-MOF, the peak at 855.6 eV with satellite peak at 860.9 eV was assigned to the Ni 2p_3/2_, and the peak at 873.0 eV with satellite peak at 880.0 eV was assigned to the Ni 2p_1/2_, which are typical peaks for the Ni. In case of NiO@C and Ni@C the Ni 2p_3/2_ is centered on 852.2 eV and 853.3 eV possible suggesting the existence of metallic Ni. Besides, the appearance of a shoulder peak at 855.33 eV for NiO@C and Ni@C suggests that some oxides of the Ni are also present in the structure.

**Table 1:** Pore volume and surface area of Ni-MOF, Ni@C, and NiO@C particles.

Samples	Carbon content (%)	BET (m^2^ g^−1^)	Pore volume (cm^3^ g^−1^)	Avg. pore size (nm)
Ni-MOF	23.2	15.8	0.037	8.5
Ni@C	27.0	122.5	0.421	12.8
NiO@C	0.2	34.2	0.155	15.6

**Figure 2:**
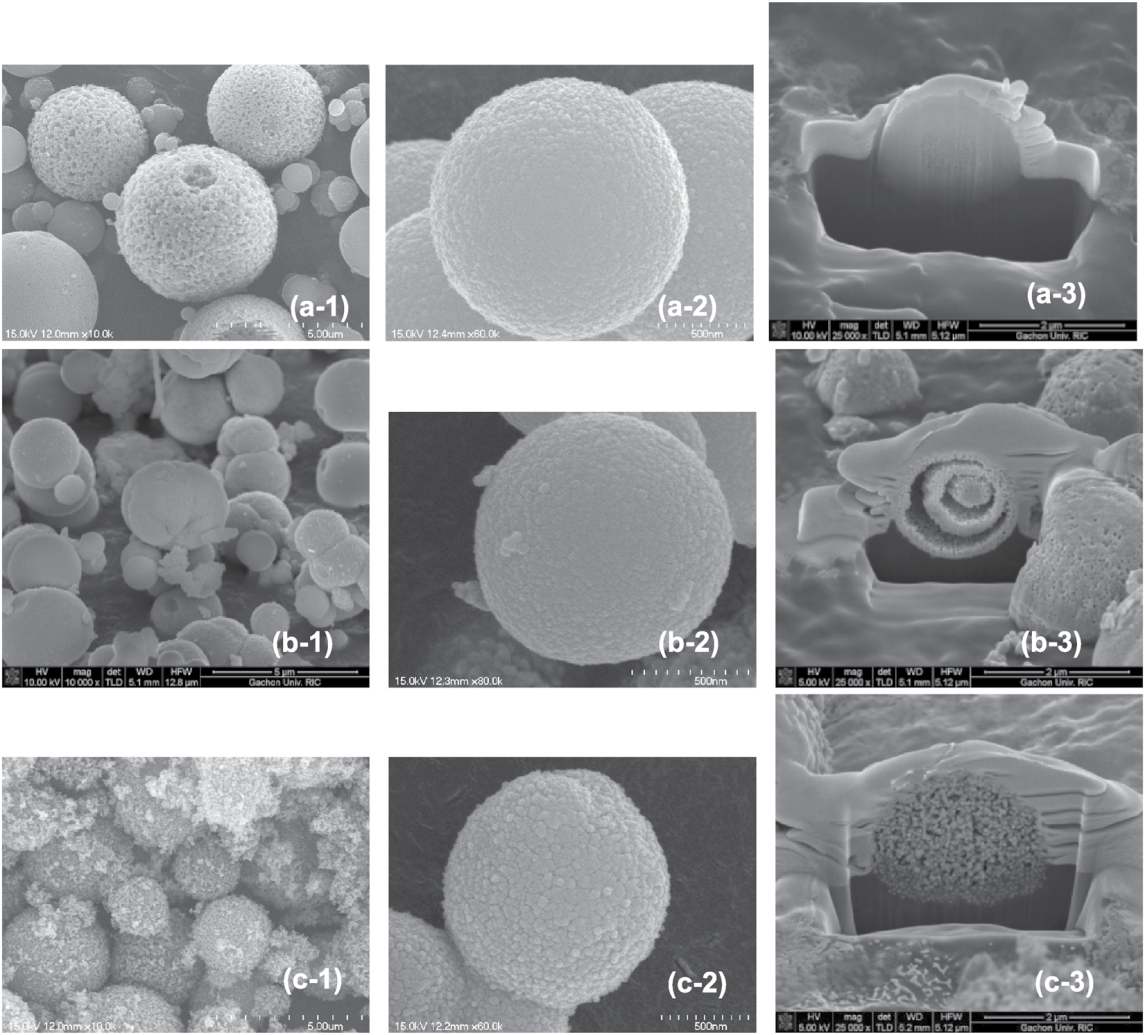
SEM (1, 2) and cross-sectional FIB-SEM (3) images of Ni-MOF (**a**), NiO@C (**b**), and Ni@C (**c**).

**Figure 3:**
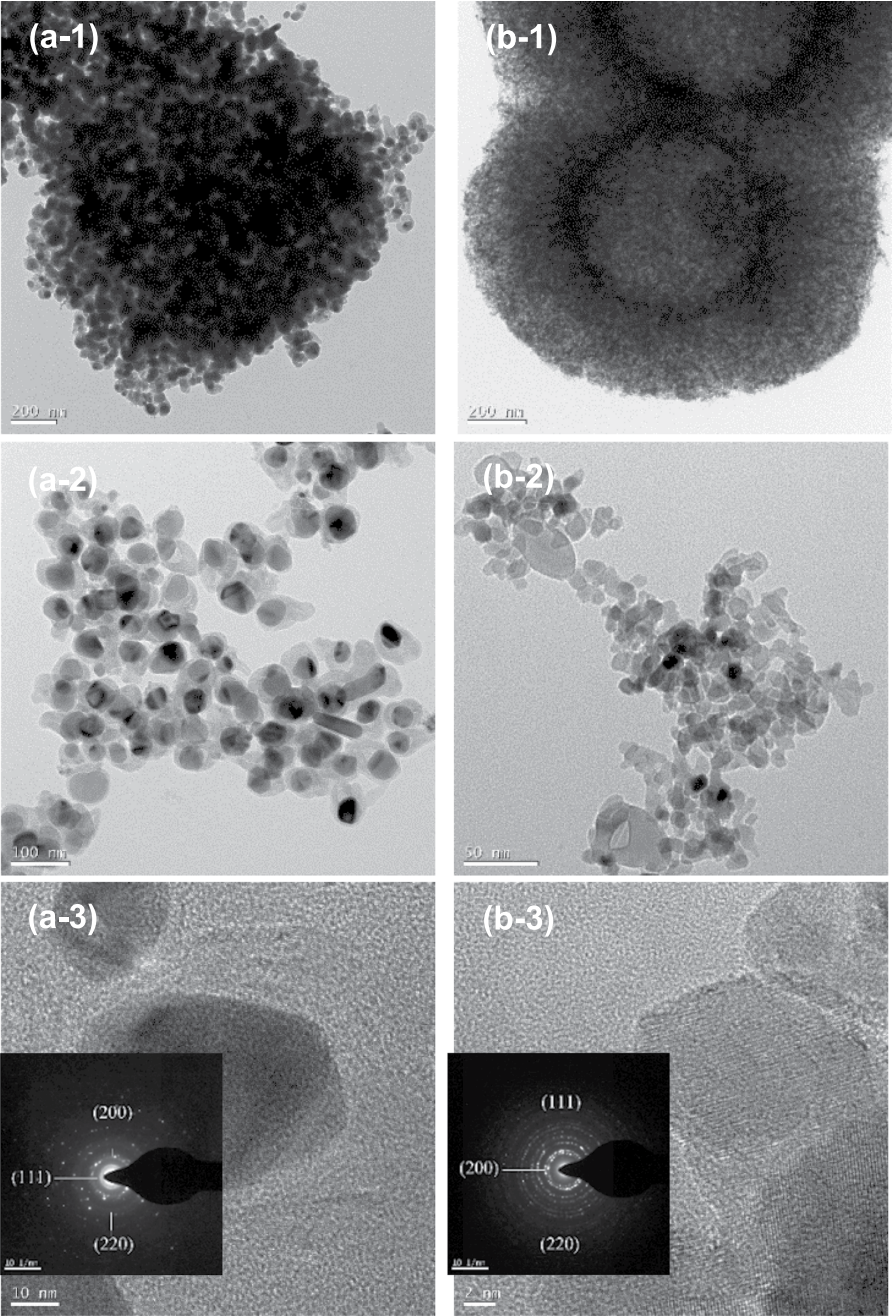
TEM (1, 2) and HRTEM (3) images of Ni@C (**a**) and NiO@C (**b**). Insets show SAED pattern.

Figure 2 shows SEM and cross-sectional FIB-SEM images of as-prepared Ni-MOF, Ni@C, and NiO@C particles, revealing that those of Ni-MOF were spherical (Fig. 2a-1,a-2) with a porous inside (Fig. 2a-3). Similarly, Ni@C and NiO@C also featured spherical particles with a more porous structure, which reflected the decomposition of organic compounds in the Ni-MOF precursor. NiO@C particles, obtained by calcination in air, exhibited a ball-in-ball structure with a crevice between the spherical core and the shell (Fig. 2b), as observed previously^26^, probably because fast mass transfer and high weight loss during calcination in air resulted in core shrinkage and the formation of a gap between the core and the shell. In contrast, Ni@C, obtained by calcination in nitrogen, retained the structure of the Ni-MOF precursor, featuring more abundant and uniformly distributed pores (Fig. 2c). The SEM images also showed that each catalyst particle comprised nano-sized subunits forming nano-sized pores. In addition, the elemental maps of both the catalysts revealed uniform distributions of Ni and C, and the elemental spectra showed that the Ni/C ratio was close to that observed by an elemental analyzer (Suppl. Fig. S3).

The structures of Ni@C and NiO@C subunits were characterized by TEM. Figure 3 clearly shows that Ni@C and NiO@C particles comprised clusters of individual uniformly distributed nanoparticles with average sizes of ~50 nm and clusters of randomly distributed nanoparticles with sizes of 10–50 nm, respectively. Interestingly, the subunit particles of Ni@C featured two distinct regions (Fig. 3a-2), i.e., a Ni-abundant core (darker area) and a carbon-abundant outer layer (lighter area)^27^. However, no clear distinct regions were observed in NiO@C subunit particles, because NiO@C contained only 0.2 wt% carbon. Therefore, the uniform distribution of fine Ni particles in Ni@C was attributed to the presence of carbon therein. Selected area electron diffraction (SAED) patterns (insets of Fig. 3a-3,b-3) confirmed the presence of NiO (JCPDF No. 65-2901)^26^ and cubic-structured Fm-3m Ni (JCPDF 65-0380)^29^ in NiO@C and Ni@C, respectively, in good agreement with XRD results.

The structural differences of Ni-MOF, Ni@C, and NiO@C resulted in different specific surface areas and porosities as shown in Table 1. Calcination of Ni-MOF considerably increased BET specific surface area and porosity, depending on calcination conditions. Ni@C, which was calcined in N_2_, had higher carbon content and higher BET surface area than NiO@C, which was calcined in air. The result might indicate that the carbon content affected the particle morphology and specific surface area of calcined products. The average pore sizes of Ni-MOF, Ni@C, and NiO@C particles were calculated from the Barrett-Joyner-Halenda (BJH) pore size distribution curve as 8–12 nm (Suppl. Fig. S4), which were indicative of mesoporosity.

### Electrocatalytic properties of Ni-MOF, Ni@C, and NiO@C

**Figure 4:**
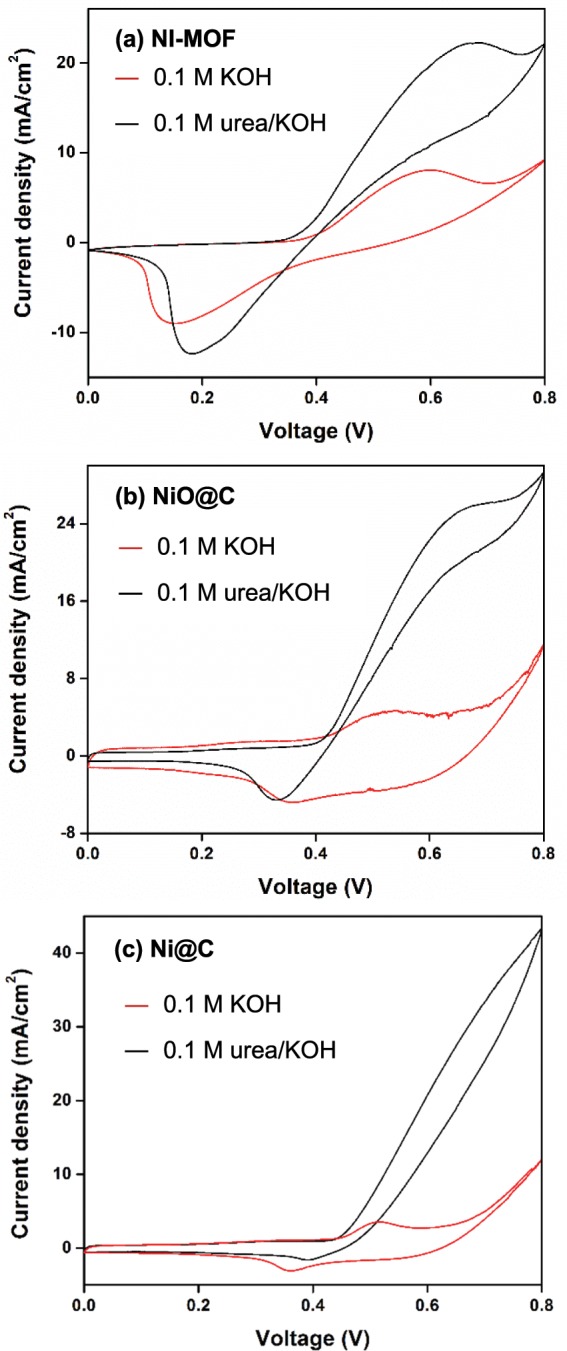
CV curves of Ni-MOF (**a**), NiO@C (**b**), and Ni@C (**c**) recorded in the absence and presence of 0.1 M urea in 0.1 M KOH at scan rate of 10 mV s^−1^.

**Figure 5:**
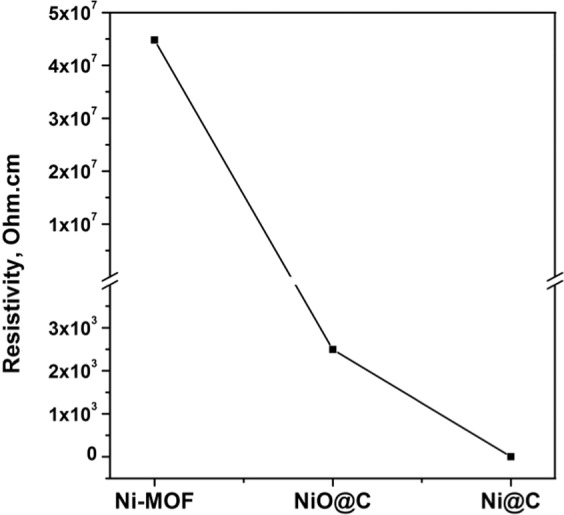
Electrical resistivity of Ni-MOF, NiO@C, and Ni@C pellets as measured by a four-point probe method.

The catalytic activities of Ni-MOF, Ni@C, and NiO@C for urea electro-oxidation were examined by cyclic voltammetry (CV) measurements (Fig. 4). All CV curves of Ni-based catalysts in the absence of urea (Fig. 4a) exhibited a pair of redox peaks at 0.5–0.6 and 0.15–0.35 V vs. Ag/AgCl, which corresponded to anodic and cathodic peak potentials, respectively, and indicated the previously reported inter-conversion of Ni^2+^/Ni^3+^ in alkaline medium^31,32^. In the presence of 0.1 mM urea in 0.1 M KOH, the Ni-based catalysts (Fig. 4b) exhibited redox peaks (with increased oxidation peak intensity and decreased reduction peak intensity) at the similar potentials as those observed in 0.1 M KOH in the absence of urea, which confirmed the activity of these catalysts for urea oxidation occurring via Ni^2+^/Ni^3+^ redox pathway; i.e., Ni^3+^OOH was formed by electrochemical oxidation of Ni^2+^(OH)_2_ and it catalyzed chemical oxidation of the adsorbed urea while being reduced to Ni^2+^OH, called an indirect electrochemical-chemical (EC) mechanism^13,33^. The result, therefore, implied that Ni^3+^OOH, a high valence of Ni oxide^34,35^, was the active site for UOR. As seen in Fig. 4, redox peak potentials were shifted by the presence of urea. Although further study should be needed for the clear explanation on the potential shift, it might be due to the reactions involving the adsorbed urea. Among the catalysts tested, Ni@C exhibited the highest peak current density of 30.3 mA cm^–2^, whereas values of 22.0 and 25.8 mA cm^−2^ were obtained for Ni-MOF and NiO@C, respectively. The results indicated that Ni@C and NiO@C derived from Ni-MOF exhibited enhanced catalytic activities for UOR compared to that of their precursor, which was mainly ascribed to their improved surface area and porosity. Furthermore, the highest electrochemical activity of Ni@C was ascribed to its high BET surface area and the co-presence of carbon, which could increase electron conductivity^26^, as evidenced by the resistivity measurement. As shown in Fig. 5, Ni@C powder pellet, which contained highest residual carbon, exhibited lowest resistivity and thus highest catalytic activity. The positive role of carbon on the catalytic performance via the enhanced conductivity was also reported previously^36^.

The electrochemical UOR on the Ni@C catalyst was appeared to be an irreversible diffusion-controlled process because the peak current density for the urea oxidation increased linearly with the square root scan rate (Suppl. Fig. S5) as observed previously^33,34^. In addition, the effect of urea and KOH concentration revealed saturation kinetics of UOR on the Ni@C catalyst (Suppl. Figs. S6 and S7).

Electrochemical active surface areas (EASAs) were calculated from the CV data (Fig. 4) as described elsewhere^21,37^. The calculation procedure and other electrochemical parameters extracted from CV data are presented in Suppl. Table S1. The EASAs of Ni-MOF, NiO@C, and Ni@C were 45.3, 55.6, and 95.1 m^2^ g^−1^, respectively, and agreed with the results of BET surface area measurements. The highest EASA of Ni@C was ascribed to the good dispersion of fine Ni active sites owing to its uniform and highly porous structure. Chronoamperometry measurements revealed that all catalysts exhibited a stable urea oxidation current for 3600 s (Suppl. Fig. S8) with the highest current observed for Ni@C being in agreement with the results of CV measurements.

### Performances of DUFCs with Ni-MOF, Ni@C, and NiO@C anode catalysts

**Figure 6:**
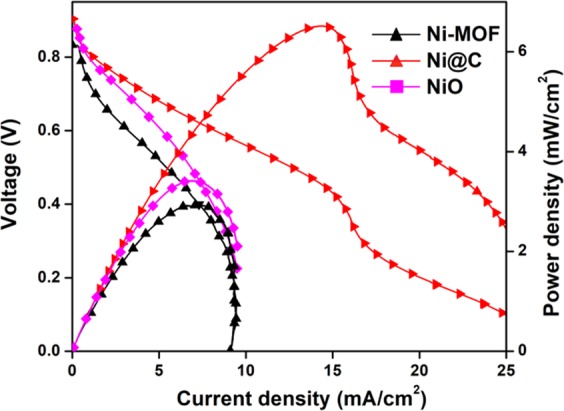
Performances DUFCs with various anode materials (Ni-MOF, NiO@C, and Ni@C) in 0.1 M urea and 1.0 M KOH at 50 °C.

DUFCs (urea/O_2_ type) were fabricated using Ni-MOF, Ni@C, and NiO@C catalysts as anode materials. The *I-V* polarization and power density curves of these cells recorded in 0.1 M urea/1.0 M KOH at 50 °C are shown in Fig. 6, and the maximum power densities (MPDs) of cells comprising Ni-MOF, NiO@C, and Ni@C anode catalysts were obtained as 2.91, 3.21, and 6.99 mW cm^−2^, respectively. The best fuel cell performance was observed for the Ni@C anode catalyst, which was mainly ascribed to its higher BET surface area and EASA, as discussed earlier.

**Figure 7:**
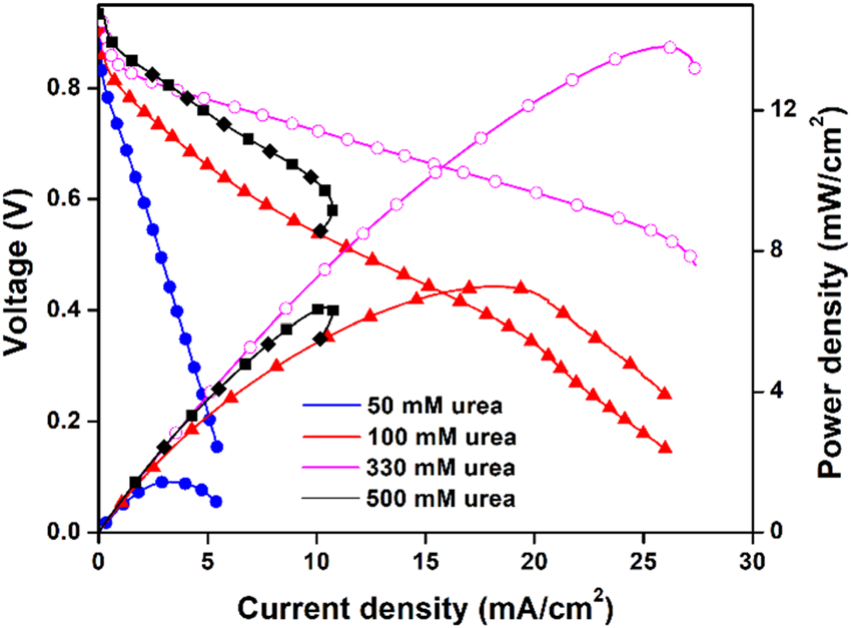
Performances of the DUFC with Ni@C as anode at different urea concentrations in 1.0 M KOH at 50 °C.

To optimize fuel cell operating conditions, the effects of urea concentration and operating temperature were studied for DUFC with Ni@C anode. As shown in Fig. 7, as the urea concentration increased in the range of 0.1–0.33 M, the MPD reached a maximum and then decreased as the urea concentration further increased, which probably reflected the deactivation and/or blockage of the catalyst at such high concentrations. At a urea concentration of 0.5 M, a transition zone (i.e., decrease of current density) was observed in the polarization curve, indicating catalyst site blockage^33,38^. The formation of the transition zone was previously attributed to the sluggish kinetics and flooding of catalyst pores by water and products of urea electro-oxidation, which prevented urea molecules from reaching the catalyst sites^39,40^.

**Figure 8:**
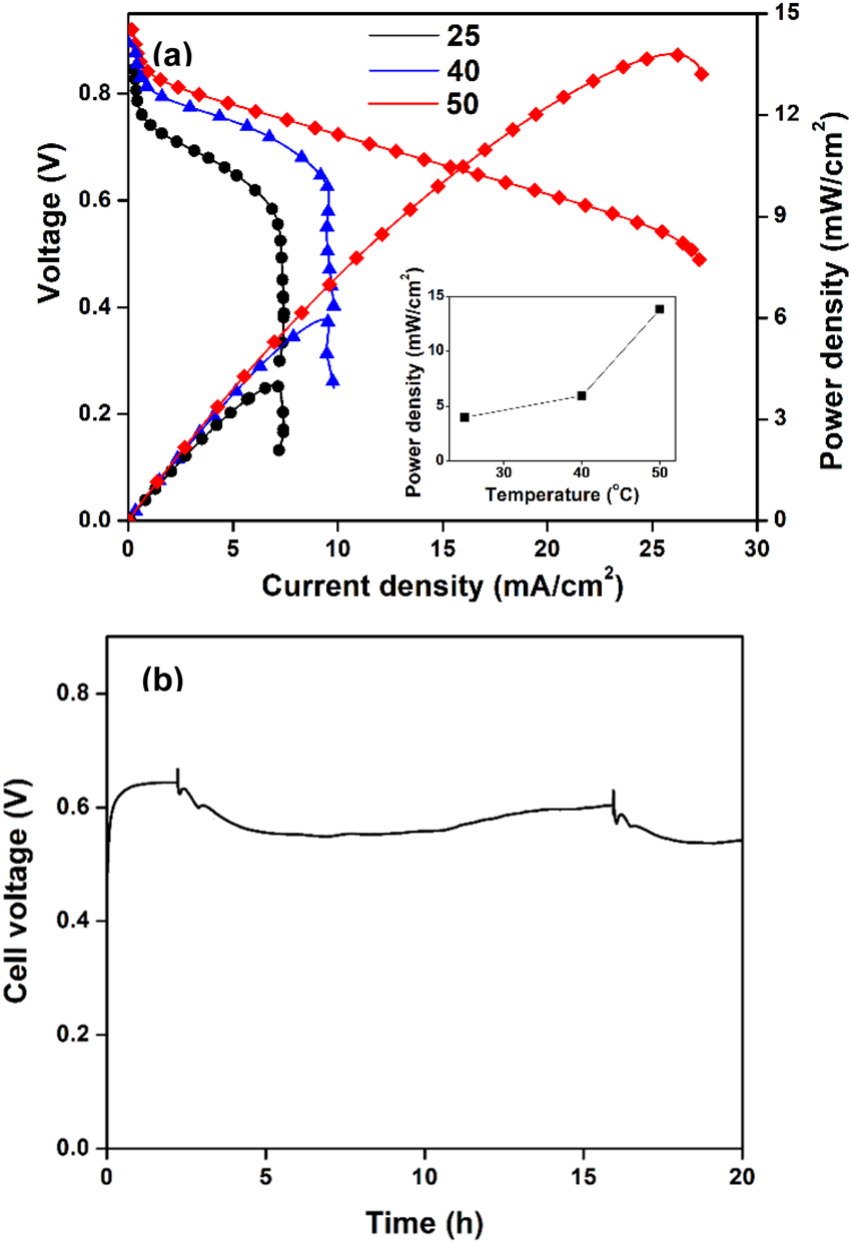
(**a**) Effect of operation temperature on the performance of DUFC with Ni@C anode in 0.33 M urea and 1.0 M KOH (Inset is peak oxidation current density vs. Temperature) and (**b**) stability test of the DUFC with Ni@C anode at a constant current density of 20 mA cm^−2^ in 0.33 M and 1.0 M KOH at 50 °C.

The performance of DUFC was also significantly affected by operating temperature, e.g., the MPD increased approximately threefold as the temperature increased from 25 to 50 °C (Fig. 8a), mainly because of the concomitant increase of electrochemical redox reaction rates and the ion conductivity of the AEM^41,42^. It should be noted that a further increase of operating temperature to values above 50 °C would enhance cell performance because CV measurements obtained for Ni@C at temperatures of 25–80 °C revealed that the peak current for urea oxidation increased continuously with temperature as shown in Suppl. Fig. S9. However, the AEM used in this study was not stable at higher temperatures, which reflected the necessity of designing a heat-stable AEM to achieve high MPD. Under optimum conditions studied (0.33 M urea/1.0 M KOH at 50 °C), the MPD of the DUFC with Ni@C anode equaled 13.8 mW cm^−2^ with an open circuit of 0.93 V, which was higher than those reported previously (Suppl. Table S2)^4–6,11,15,19,29,43–47^. The excellent performance of the Ni@C anode was mainly attributed to its high specific surface area and mesoporous structure, which provided a high number of active Ni-catalyst sites and allowed fast mass transfer of urea and products in the anode. A high MPD of 31.5 mW cm^−2^ in urea/H_2_O_2_ type fuel cell at 70 °C using 0.5 M urea/7.0 M KOH as anolyte, and 2 M H_2_O_2_ and 2 M H_2_SO_4_ as catholyte has been reported^4^. However, the operation with H_2_O_2_ and H_2_SO_4_ as catholyte chemicals should require a higher cost in comparison to the humidified air.

The stability of the DUFC with Ni@C anode was further examined under a constant current density of 20 mA cm^−2^ in 0.33 M urea/1.0 M KOH at 50 °C (Fig. 8b). Notably, the cell voltage was retained at ~0.6 V during 15 h of operation, slightly decreasing and remaining constant until 20 h.

## Conclusion

Highly porous nanostructured Ni@C and NiO@C composites synthesized by simple calcination of a Ni-based MOF exhibited higher specific surface areas and EASAs than their Ni-MOF precursor and thus featured excellent electro-catalytic activity for urea electro-oxidation. A DUFC containing Ni@C as an anode catalyst achieved a maximum power density of 13.8 mW cm^−2^ in 330 mM urea/1 M KOH at 50 °C, outperforming previously reported DUFCs. Moreover, the above fuel cell exhibited a stable performance during 20 h of continuous operation. In view of the above, it was concluded that the simply prepared nanostructured Ni@C and NiO@C catalysts are suitable anode materials for DUFCs.

## Methods

### Syntheses of Ni-MOF, Ni@C, and NiO@C

Ni@C and NiO@C were synthesized using Ni-MOF as a template precursor. To prepare Ni-MOF ([Ni_3_(btc)_2_], btc = benzene-1,3,5-tricarboxylate), a solution of Ni(NO_3_)_3_ · 6H_2_O in methanol was mixed with an appropriate amount of benzene-1,3,5-tricarboxylic acid, stirred for 1 h at room temperature, and autoclaved at 150 °C for 24 h. After reaction completion, the suspension was centrifuged, and the solid product was repeatedly washed with methanol and dried in a vacuum oven at 60 °C for 24 h. The thus obtained Ni-MOF precursor powder was calcined at 400 °C (5 °C min^−1^) for 4 h in nitrogen or air to synthesize Ni@C or NiO@C, respectively.

### Preparation of electrodes and urea/O_2_ single cell

As prepared Ni-MOF, Ni@C, and NiO@C catalysts were mixed with Vulcan carbon (XC-72) at 30 wt%, and the obtained mixture was added into 0.05 wt% Nafion solution in isopropanol and sonicated for 2 h. The resulting anode catalyst inks were coated onto a nickel foam (pore diameter 0.25 mm, MTI, USA) electrode to achieve a catalyst loading of 3.4 mg cm^−2^. A commercial Pt/C (40 wt%, E-TEK)-coated carbon paper with a Pt loading of 0.6 mg cm^−2^ was used as a cathode. An anion exchange membrane (AEM; Fumasep FAA-3-PK-130, Germany) was used as a polymer electrolyte separating anode and cathode compartments, as described elsewhere^48^. The active electrode area equaled 5.0 cm^2^. A single-cell bipolar plate was prepared using graphite with serpentine flow channels. Urea solutions were pumped into the anode side by a peristaltic pump at 3 mL min^–1^, and humidified air was supplied to the cathode by passing air through room-temperature water.

### Analysis

Crystal structures were analyzed by X-ray diffraction (XRD; Rigaku DMAX 2200, Japan) at a scan rate of 5° min^−1^ using Cu *K*_α_ radiation (*λ* = 1.506 Å). Sample morphology and structure were probed by scanning electron microscopy (SEM; Hitachi S-4700, Japan), focused ion-beam SEM (FIB-SEM; Hitachi, Japan), and transmission electron microscopy (TEM; Tecnai G2 F30 S-Twin, USA). Thermogravimetric (TG) analysis was performed using a TG analyzer (SDT Q600, TA, USA). Carbon contents were measured by an elemental analyzer (Vario MICRO cube, Germany). Specific surface area and porosity were determined based on nitrogen adsorption-desorption measurements performed using a Micromeritics ASAP 2020 apparatus according to the Brunauer-Emmett-Teller (BET) theory. Electrical resistivity of the catalyst powder compressed into pellets under 1500 bar at 25 °C in 30 minutes was determined by four-probe resistivity method (CMT-SR2000N, South Korea). The electrochemical properties of catalysts were analyzed by a potentiostat-galvanostat (VSP, Bio-Logic Science Instruments, France) using a conventional three-electrode system with Pt wire and Ag/AgCl (3 M NaCl) as counter and reference electrodes, respectively. The working electrode was prepared by coating catalyst inks (as described above) on carbon fiber (5 cm^2^) to achieve a catalyst loading of 1.0 mg cm^−2^. Fuel cell performance was evaluated utilizing the potentiostat-galvanostat interfaced with EC-lab 11.01 data acquisition software.

## Supplementary information

Supplemental information.

## Data Availability

The datasets generated during and/or analysed during the current study are available from the corresponding author on reasonable request.
