# Supplementary material for: Metal–organic framework–derived Ni@C and NiO@C as anode catalysts for urea fuel cells
Source: Sci Rep. 2020 Jan 14;10:278. doi: 10.1038/s41598-019-57139-7 (PMC6959365; doi:10.1038/s41598-019-57139-7)
Supplement: Supplementary file 1 — Supplemental information. [file 41598_2019_57139_MOESM1_ESM.pdf]

Electronic Supplementary Information for:

Metal–organic framework–derived Ni@C and NiO@C as anode catalysts for urea fuel cells

Thao Quynh Ngan Tran, Bang Ju Park, Woo Hyeon Yoon, Tien Nhac Duong & Hyon Hee Yoon*

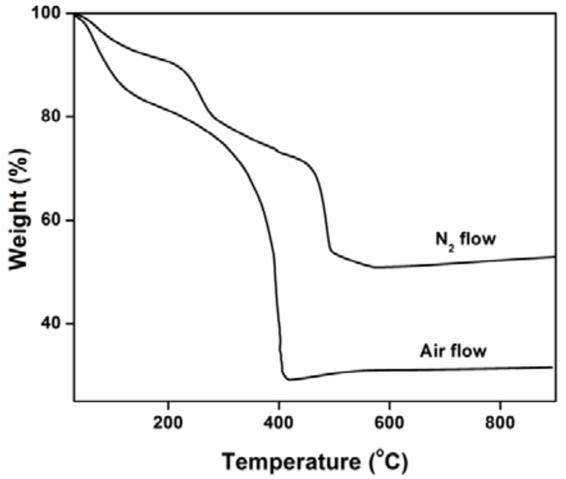

Fig. S1. TG analysis curves of Ni-MOF in air and nitrogen

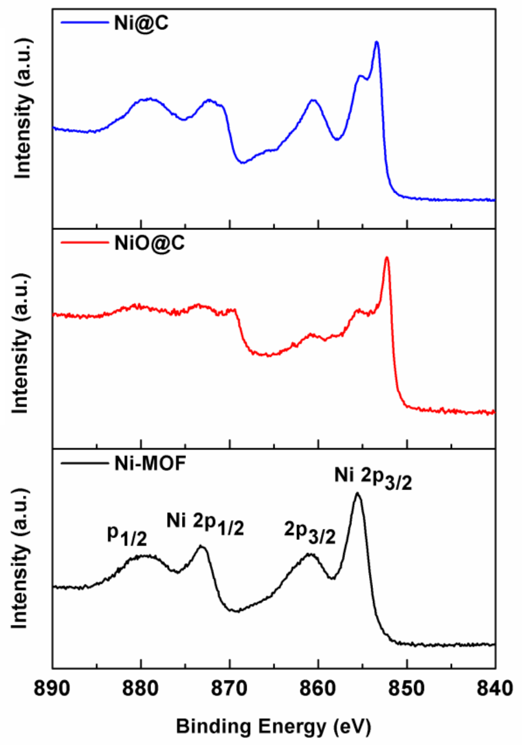

Fig. S2. High resolution Ni 2p peak of Ni-MOF, NiO@C, and Ni@C

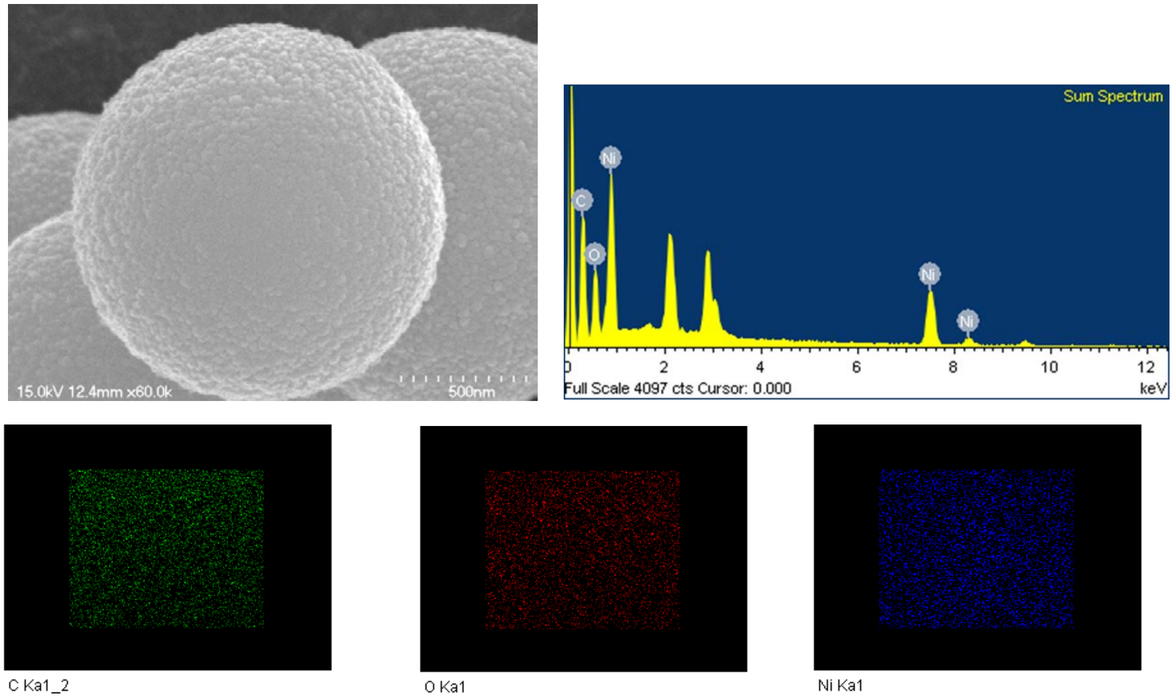

Fig. S3-a. SEM, EDX spectra, and elemental mapping of Ni-MOF.

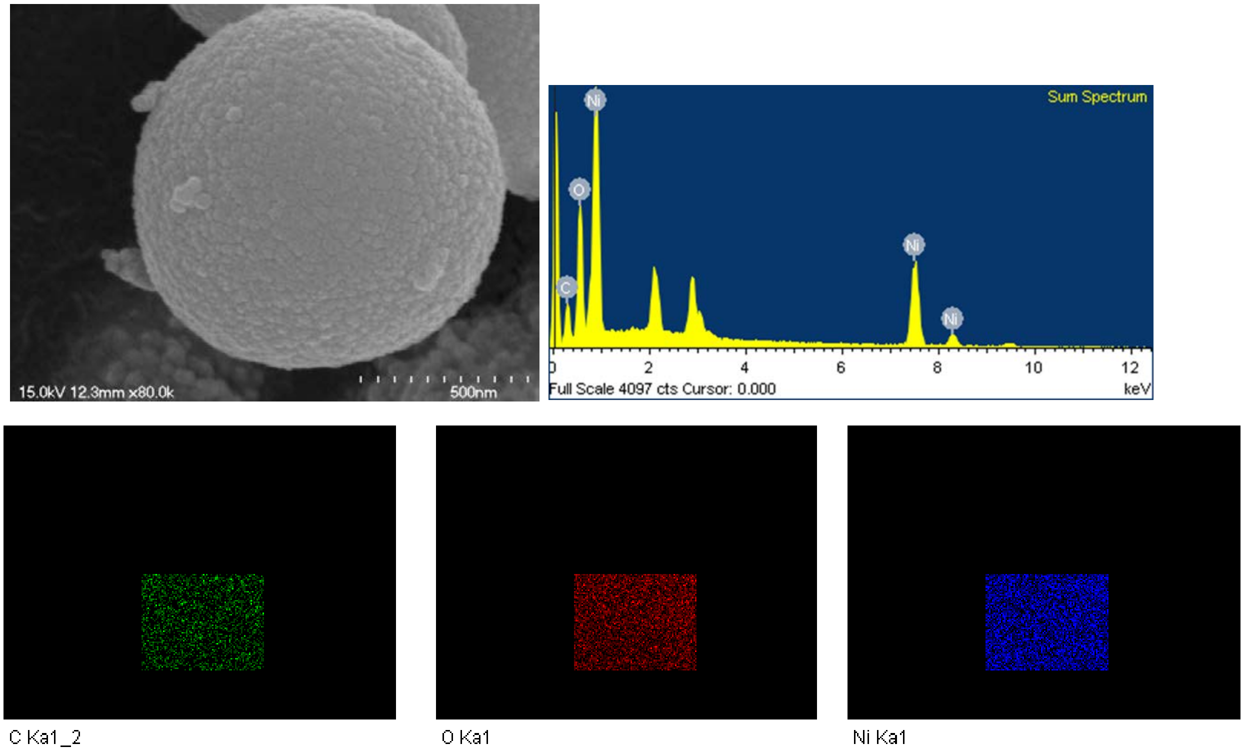

Fig. S3-b. SEM, EDX spectra, and elemental mapping of NiO@C

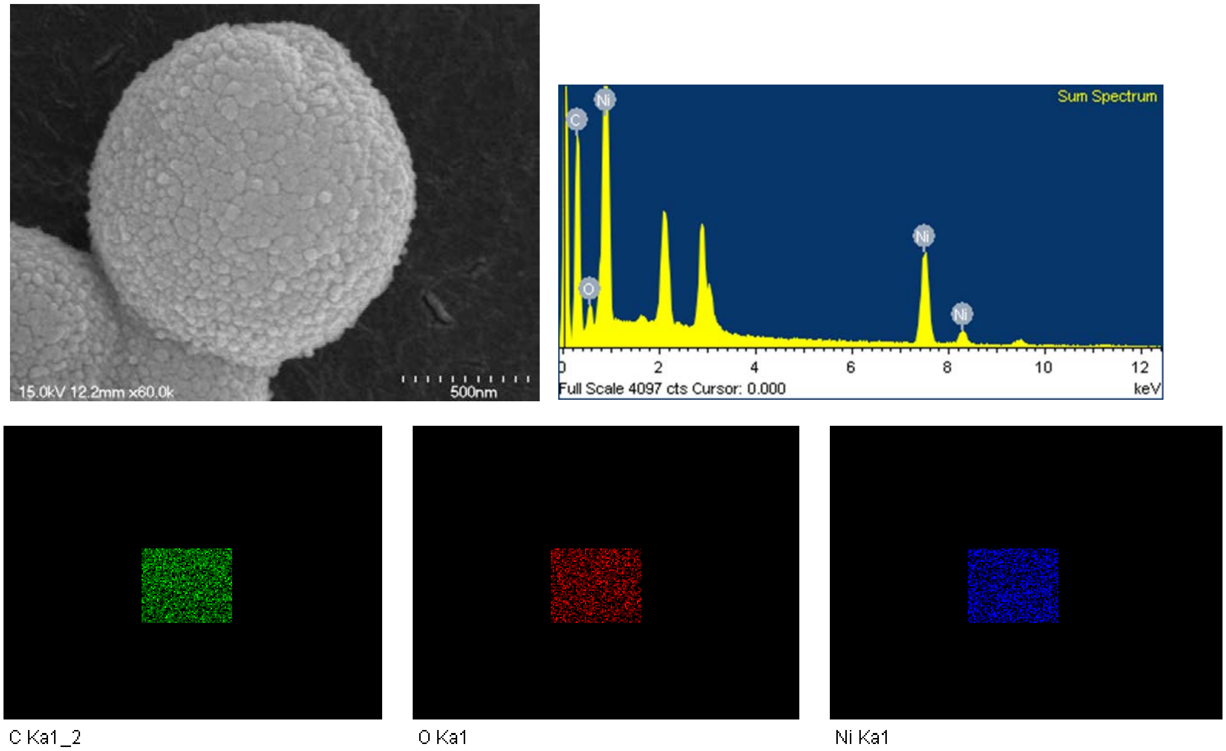

Fig. S3-c. SEM, EDX spectra, and elemental mapping of NiO@C

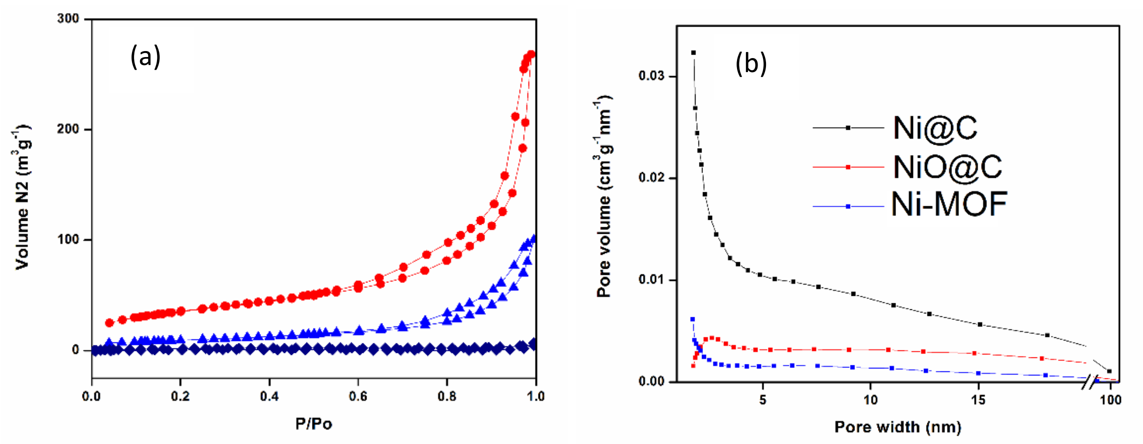

Fig. S4. (a) N_2 adsorption isotherms and (b) the pore diameter distribution of Ni-MOF, NiO@C, and NiO@C particles.

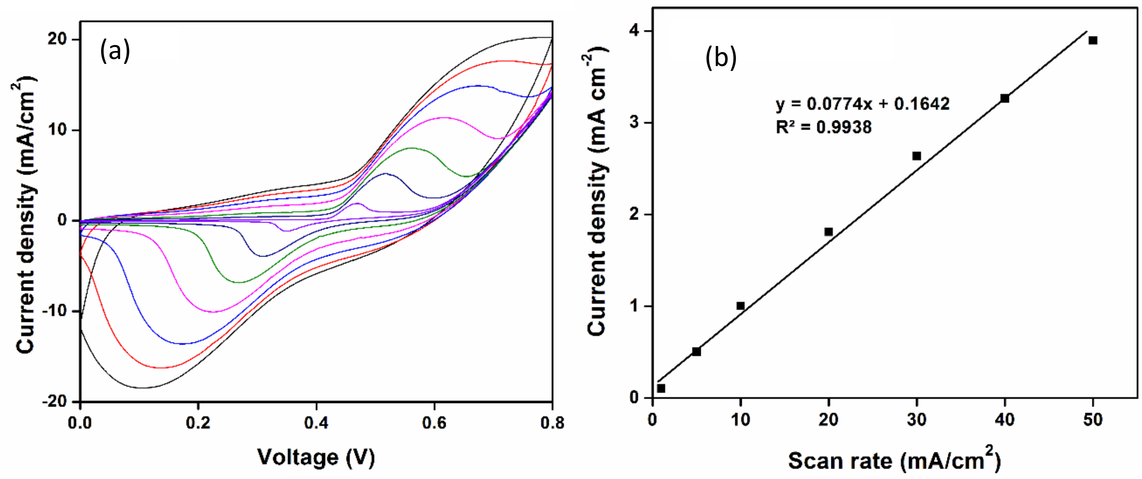

Fig. S5. (a) CV curves of Ni@C at different scan rates in 10mM urea and 0.1M KOH solution. (b) the anodic peak currents vs. the square root of scan rate.

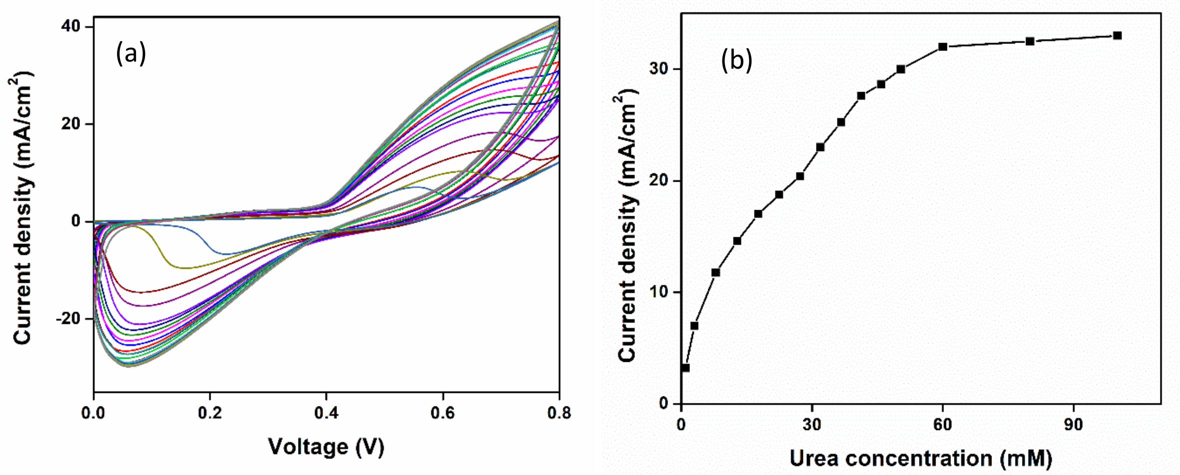

Fig. S6. (a) CV curves of Ni@C at different urea concentration in 0,1M KOH. (b) effect of urea concentration on peak current density and potential.

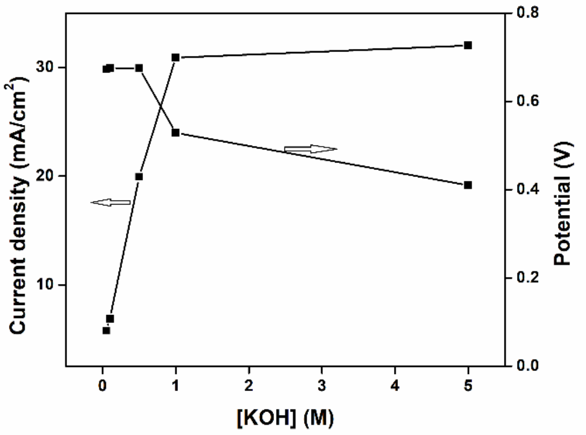

Fig. S7. The effect of KOH concentration in 100 mM urea on peak current density and potential of Ni@C and 10mV/s of scan rate.

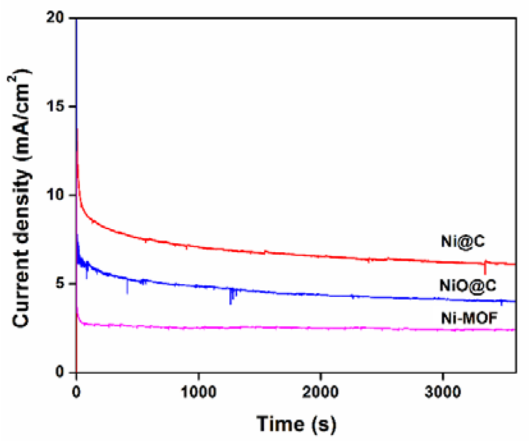

Fig. S8. Chronoamperometry plot of Ni-MOF, NiO@C, and Ni@C catalysts in 100 mM urea in 0.1 M KOH at 0.55 V.

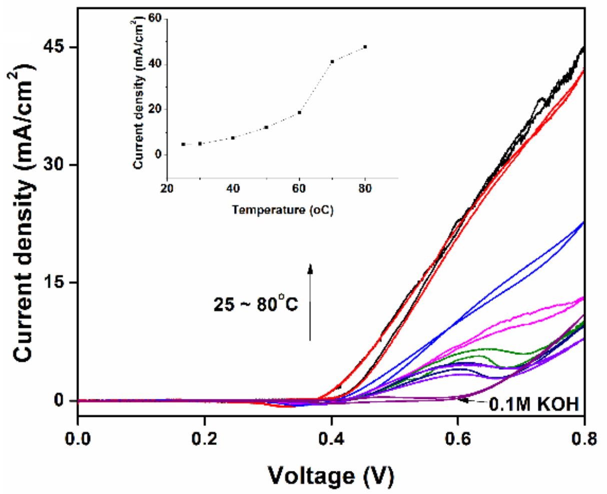

Fig. S9. CV curves of Ni@C at different temperatures. Inset is peak oxidation current density vs. temperature.

Table S1. Comparison of electrochemically surface area of different electrode materials.

Samples	ECSA[a)] (m^2 g^{-1})	Mass activity [b)] (mA mg^{-1}Ni)	Specific activity [c)] (mA cm^{-2} Ni)
**Ni@C**	95.13	27.67	0.29
**NiO@C**	55.64	21.93	0.39
**Ni-MOF**	45..28	25.98	0.57

a) The electrochemically active surface area (ECSA) was estimated according to the following equation: ESCA = $Q/(m \cdot q)$, where Q is the charge required to reduce Ni^{3+} to Ni^{2+}, m is the

loading of Ni, and q is 257 μC cm^{-2} for the one electron process in the conversion of NiOOH to $Ni(OH)_2$ [1,2]. Q was calculated by integrating the peak from the CV curves.

b) Mass activity was calculated by dividing the anodic peak current by the Ni loading.

c) Specific activity was estimated by dividing the mass activity by the ESCA.

Table S2. Comparison of reported performances of urea fuel cells.

Anode	**Fuel**	**Cathode**	**Oxidant**	**OCV, V**	**MPD[1)], $mWcm^{-2}$**	**Ref.**
Ni/C	1 M urea, 50°C	Mn/C	Wet air	0.65	1.7	[3]
NiPT/C	1 M urea, 60°C	MnO_2/C	Wet air	0.83	14.2	[4]
Ni/ MWCNT	1 M urea/1.5 M NaOH	Pt/C	20% H_2O_2 +5% H_3PO_4	0.262	0.056	[5]
Ni_4Co_1/C	0.33 M urea, 60°C	Pt/C	Wet air	0.65	2.1	[6]
RuO_2	30%wt Urea, 300°C	Pt/N-doped graphene	Wet air, 90°C	0.5	26.5	[7]
Ni/C	Fresh urine	carbon cloth	300 mg L^{-1} Cr(VI) + 0.5 M H_2SO_4	1.21	2.5	[8]
$Ni_{0.2}Co_{0.8}$/Ni foam	0.33 M Urea 5 M KOH, 70°C	Pd/C	2 M H_2O_2 + 2 M H_2SO_4	0.88	31.5	[9]
Ni-Co	0.33 M urea 9 M KOH, RT	Pd/C	H_2SO_4 + H_2O_2	0.92	7.4	[10]
Ni/C	1 M urea 8 M NaOH, RT	Pd/C	1M H_2O_2 + 2M H_2SO_4	1.33	12.3	[11]
Ni/ graphene	0.33 M Urea 2M KOH, RT	Pt/C	Air	0.197	4.06	[12]
SNF–MWCNT	0.33 M Urea 0.1 M KOH, 55°C	Pt/C	Wet air	0.9	0.41	[13]
Ni/CNT	3 M urea, 3 M KOH, 55°C	Pt/C	1.5 M H_2SO_4	0.96	<6.6	[14]
Ni@C	0.33 M urea, 1 M KOH, 50°C	Pt/C	Wet air	0.93	13.82	This work

References

[1] G. Das, R.M. Tesfaye, Y. Won, H.H. Yoon, NiO-Fe_2O_3 based graphene aerogel as urea electrooxidation catalyst, Electrochim. Acta. 237 (2017) 171–176.

[2] W. Yan, D. Wang, G.G. Botte, Electrochemical decomposition of urea with Ni-based catalysts, Appl. Catal., B. 127 (2012) 221–226.

[3] R. Lan, S. Tao, J.T.S. Irvine, A direct urea fuel cell – power from fertiliser and waste, Energy Environ. Sci. 3 (2010) 438–441.

[4] R. Lan, S. Tao, Preparation of nano-sized nickel as anode catalyst for direct urea and urine fuel cells, J. Power Sources. 196 (2011) 5021–5026.

[5] E.C. Serban, A. Balan, A.M. Iordache, A. Cucu, C. Ceaus, M. Necula, G. Ruxanda, C. Bacu, E. Mamut, I. Stamatin, Urea/hydrogen peroxide fuel cell, J. Nanomater. Bios. 9 (2014) 1647–1654.

[6] W. Xu, H. Zhang, G. Li, Z. Wu, Nickel-cobalt bimetallic anode catalysts for direct urea fuel cell, Sci.

Rep. 4 (2014) 5863.
[7] M. Nagao, K. Kobayashi, T. Hibino, A Direct Urine Fuel Cell Operated at Intermediate Temperatures, Chem. Lett. 44 (2015) 363–365. doi:10.1246/cl.141067.
[8] W. Xu, H. Zhang, G. Li, Z. Wu, A urine/Cr(VI) fuel cell - Electrical power from processing heavy metal and human urine, J. Electroanal. Chem. 764 (2016) 38–44.
[9] F. Guo, D. Cao, M. Du, K. Ye, G. Wang, W. Zhang, Y. Gao, K. Cheng, Enhancement of direct urea-hydrogen peroxide fuel cell performance by three-dimensional porous nickel-cobalt anode, J. Power Sources. 307 (2016) 697–704.
[10] F. Guo, K. Cheng, K. Ye, G. Wang, D. Cao, Preparation of nickel-cobalt nanowire arrays anode electro-catalyst and its application in direct urea/hydrogen peroxide fuel cell, Electrochim. Acta. 199 (2016) 290–296.
[11] Z. Fan, Y. Kwon, X. Yang, W. Xu, Z. Wu, In-situ production of hydrogen peroxide as oxidant for direct urea fuel cell, Energy Procedia. 105 (2017) 1858–1863.
[12] A. Yousef, M.H. El-Newehy, S.S. Al-Deyab, N.A.M. Barakat, Facile synthesis of Ni-decorated multi-layers graphene sheets as effective anode for direct urea fuel cells, Arab. J. Chem. 10 (2017) 811-822.
[13] N. Kakati, J. Maiti, K.S. Lee, B. Viswanathan, Y.S. Yoon, Hollow Sodium Nickel Fluoride Nanocubes Deposited MWCNT as An Efficient Electrocatalyst for Urea Oxidation, Electrochim. Acta. 240 (2017) 175–185.
[14] H. Zhang, Y. Wang, Z. Wu, D.Y.C. Leung, A direct urea micro fl uidic fuel cell with fl ow-through Ni-supported- carbon- nanotube-coated sponge as porous electrode, J. Power Sources. 363 (2017) 61–69.
